# Implementation of a malaria rapid diagnostic test in a rural setting of Nanoro, Burkina Faso: from expectation to reality

**DOI:** 10.1186/s12936-018-2468-1

**Published:** 2018-08-30

**Authors:** Francois Kiemde, Marc Christian Tahita, Massa dit Achille Bonko, Petra F. Mens, Halidou Tinto, Michael Boele van Hensbroek, Henk D. F. H. Schallig

**Affiliations:** 1 Institut de Recherche en Science de la Sante-Unité de Recherche Clinique de Nanoro, Nanoro, Burkina Faso; 2 Parasitology Unit, Department of Medical Microbiology, Academic Medical Centre, Amsterdam, The Netherlands; 3 Global Child Health Group, Academic Medical Centre, University of Amsterdam, Amsterdam, The Netherlands

**Keywords:** Malaria, HRP-2, RDT, Microscopy, Sensitivity and specificity

## Abstract

**Background:**

Malaria rapid diagnostic tests (RDTs) are nowadays widely used in malaria endemic countries as an alternative to microscopy for the diagnosis of malaria. However, quality control of test performance and execution in the field are important in order to ensure proper use and adequate diagnosis of malaria. The current study compared the performance of a histidine-rich protein 2-based RDT used at peripheral health facilities level in real life conditions with that performed at central reference laboratory level with strict adherence to manufacturer instructions.

**Methods:**

Febrile children attending rural health clinics were tested for malaria with a RDT provided by the Ministry of Health of Burkina Faso as recommended by the National Malaria Control Programme. In addition, a blood sample was collected in an EDTA tube from all study cases for retesting with the same brand of RDT following the manufacturer’s instructions with expert malaria microscopy as gold standard at the central reference laboratory. Fisher exact test was used to compare the proportions by estimating the p-value (p ≤ 0.05) as statistically significant.

**Results:**

In total, 407 febrile children were included in the study and malaria was diagnosed in 59.9% (244/407) of the cases with expert malaria microscopy. The sensitivity of malaria RDT testing performed at health facilities was 97.5% and comparable to that achieved at the laboratory (98.8%). The number of malaria false negatives was not statistically significant between the two groups (p = 0.5209). However, the malaria RDT testing performed at health facilities had a specificity issue (52.8%) and was much lower compared to RDT testing performed at laboratory (74.2%). The number of malaria false positives was statistically significantly different between the two groups (p = 0.0005).

**Conclusion:**

Malaria RDT testing performed at the participating rural health facilities resulted in more malaria false positives compared to those performed at central laboratory. Several factors, including storage and transportation conditions but also training of health workers, are most likely to influence test performance. Therefore, it is very important to have appropriate quality control and training programmes in place to ensure correct performance of RDT testing.

## Background

The National Malaria Control Programme (NMCP) guidelines in Burkina Faso recommend that all suspicious malaria cases should be confirmed using either a RDT or light microscopy (if available) [1, 2]. Microscopy detecting *Plasmodium* parasites in Giemsa-stained thick or thin blood slides still remains the gold standard for malaria diagnosis [3]. The sensitivity and specificity of microscopy is however depending on the quality of the blood films, maintenance of the microscopy and training of the microscopists. Therefore, in many peripheral health settings RDTs have been introduced to fill this gap [4]. Malaria RDTs are in principle easy to perform in the field outside of a conventional laboratory, do not need much training to be performed, are relative cheap, and give a diagnostic result within 15 min [5].

The decision of many countries of sub-Saharan Africa (SSA), including Burkina Faso, to select malaria rapid diagnostic test detecting *Plasmodium falciparum*-specific histidine-rich protein 2 (*Pf*HRP2) for diagnosis malaria is based on the high sensitivity and specificity reported by the World Health Organization (WHO) and Foundation for Innovative New Diagnostics (FIND) malaria RDT evaluation programme [6]. Secondly, HRP2-based RDTs are reported to have a good thermal and humid stability compared to tests targeting *Plasmodium*-specific parasite lactate dehydrogenase (pLDH) [7]. Indeed, exposure of *Pf*HRP2 RDT to high temperature (up to 45°C) over a prolonged period of up to 24 months did not affect the quality of the test [6]. However, the influence of a very high temperature (> 45 °C; which is common in several SSA countries) and humidity (> 65%) are not documented. In addition, transport and storage conditions and operator performance have been reported to influence RDT performance [8–11]. Thirdly, the issue of persisting HRP2 antigens after successful treatment has been raised as a major factor contributing to reduce the specificity of *Pf*HRP2-based RDTs [12–19]. Finally, there is also increasing concern with respect to reported false positive diagnosis by *Pf*HRP2 RDT in particular in low malaria transmission settings [20].

These concerns warrant close monitoring of the performance of RDTs under field conditions. The objective of the present study was to assess the performance of the recommended HRP2 RDT by the Burkina Faso NMCP executed at peripheral level compared with the performance of the same brand of RDT in controlled conditions at a central reference laboratory with strict adherence to the manufacturer instructions.

## Methods

### Study design

The study was conducted between April and October 2016 in the health district of Nanoro, which is located at approximately 100 km from Ouagadougou, the capital city of Burkina Faso. Malaria is endemic with the transmission peak occurring between July and November and *P. falciparum* is the predominant malaria parasite [21]. The study was conducted as part of a large project aiming to assess fever aetiologies in Nanoro [22]. Briefly, children under 5 years with an axillary temperature ≥ 37.5 °C presenting at one of the participating health facilities were asked to participate. After obtaining the consent from parent or legal guardian, the participant was enrolled in the study. The malaria RDT used to screen febrile children at recruitment in the health facilities during the study period was the HRP2-based RDT specific to *P. falciparum* (SD Ag Bioline *Pf*: Standard Diagnostics, Hagal-Dong, Korea). Information on lot number and expiration date was not collected. The result of malaria RDT testing in the health facilities was recorded on a case record form.

After inclusion, a blood sample was collected for each child in ethylene diamine tetra acetic acid (EDTA) tube, transported under cold conditions in an ice-box at the laboratory of Clinical Research Unit of Nanoro (CRUN). Expert malaria microscopy was performed by expert laboratory technician from blood collected in EDTA tube before stored at − 20 °C until retesting. The retesting is done at the laboratory of CRUN with experimented technician with a *Pf*HRP2 RDT of the same manufacturer (SD Ag Bioline *Pf*: Standard Diagnostics, Hagal-Dong, Korea: Lot number: 05EDC002A; Expiration date: 01/03/2019). The RDTs are transported and stored according to the manufacturer’s instructions. Standard Operating Procedures (SOPs) for ordering, transportation, storage and performing malaria RDTs are in place at CRUN. Blood samples are thawed at room temperature before retesting.

### Laboratory procedures

For retesting in the CRUN laboratory, the blood sample was thawed at room temperature and the diagnostic test was performed according to the manufacturer’s instructions. One trained technician performed the RDT, but the result was read by two technicians and in case of a discordant opinion a third reader would be consulted. The laboratory technicians who repeated the malaria RDT were blinded from the malaria RDT results obtained at the health facilities and the RDT test results were reported on separate case record forms.

Malaria slide reading was performed by expert microscopists who are participating in an external quality programme and only certified microscopists were allowed to read the slides. The limit of detection (LoD) of this expert microscopy was 10 parasites per µl [23]. Thin films were fixed with methanol and blood slides were stained with 3% Giemsa solution (pH 7.2) for identification and quantification of asexual *P. falciparum* and other *Plasmodium* species. Parasites densities were determined by counting the number of asexual parasites per 200 white blood cells, and calculating per μl of blood by assuming the number of white blood cells to be at 8000 per μl. Thick blood smears were considered negative when the examination of 200 fields per thick film did not reveal the presence of any asexual parasites. Each blood slide was read by two independent expert readers, and in case of discordance (positive *vs* negative, different in *Plasmodium* species, difference in parasite density > Log10 or ratio > 2 in case of parasite density ≤ 400/µl or > 400/µl, respectively), the blood slide was read by a third independent reader. Positive microscopy results were recorded as the geometric means of the two reader’s results or the geometric means of the two geometrically closest reading in case of third reading. These results were expressed as asexual parasites per μl by using the patient’s white blood cell (WBC) count. A selection of slides (5%) was re-read by an independent expert microscopist for quality assurance. All microscopists were blinded from the results obtained with the different malaria RDTs.

### Ethical approval

The study was approved by the National Ethical Committee in Health Research, Burkina Faso (Deliberation N°2014-11-130). The study was also approved by the health district authorities and community leaders of different villages before implementation.

### Data analysis

Double entered data was done using Excel 2016. The data analysis was done with R software version 3.3.1 (R Foundation for Statistical Computing, Vienna, Austria). For the quantitative data, the descriptions were performed by using mean or median respectively. Proportion was used to describe qualitative data. The evaluation of the performance of the malaria RDT performed by nurses at health centres level was done by comparing the results of *Pf*HRP2 tests performed in the field by health facilities nurses to those repeated in CRUN laboratory by trained technicians. Proportions were used to present the concordance and discordance between the two tests performed in malaria positive and negative groups. Fisher exact test was used to compare the proportions by estimating the p-value (p ≤ 0.05) as statistically significant. Agreement between each RDT test and microscopy and between the two RDTs tests was determined by calculating Kappa (κ) values with 95% confidence intervals by using GraphPad software (https://www.graphpad.com/quickcalcs/).

## Results

### Description of the study population

In total, 407 children were included in the study. The median age of enrolled children was 23.0 months [IRQ (interquartile): 12.0–36.0] and the mean axillary temperature was 37.7 °C (standard deviation: 0.78 °C). Males represented 56.8% (231/407) of the study population (Table 1).

**Table 1:** Baseline characteristic of study population

Characteristics	N = 407
Age in months, median (IQR)	23.0 (12.0–36.0)
Male, n (%)	231 (56.8)
Axillary temperature °C, mean (SD)	37.7 (0.78)
Parasites/μl, geometric mean (min–max)	22,839. 4 (32–586,250)
Malaria positive by expert microscopy, n (%)	244 (59.9)
Malaria positive health facilities RDT-*Pf*HRP2, n (%)	315 (77.4)
Malaria positive laboratory RDT-*PfHRP2*, n (%)	282 (69.3)

### Results of RDT testing and expert microscopy

The number of positive cases of malaria determined by performing a *Pf*HRP2 RDT at the health facilities level by nurses (HF-*Pf*HRP2) was 77.4% (315/407). No test failures were reported at health facilities. Retesting the collected blood samples in the laboratory of CRUN (Lab-*Pf*HRP2) revealed that 69.3% (282/407) was RDT positive (Table 1). No invalid tests were observed at CRUN and there was no need for a third opinion as all initial readings were in agreement.

The number of *P. falciparum* malaria microscopy positive slides as assessed by expert microscopists was 59.9% (224/407), with a geometric mean and median parasite density of 22,839.4 parasites/µl (range 32–586,250 parasites/µl) and 39,847 (7828–95,369), respectively. Co-infections were found in 5 cases: there were 4 co-infections with *Plasmodium malariae* and 1 case with *Plasmodium ovale*. There was no need for a third reader as the two expert technicians agreed on the initial microscopy result.

### Performance of malaria RDT detecting PfHRP2 performed at the health facilities level by nurses and at the laboratory level by trained technicians compared to expert microscopy

The number of malaria true positive cases (by considering expert microscopy as the gold standard) was 58.5% (238/407) with the *Pf*HRP2 RDT supplied by the NMCP and performed by the nurses at health facilities level (HF-*Pf*HRP2) and 59.0% (240/407) with the *Pf*HRP2 RDT purchased by CRUN and performed by trained laboratory technicians at laboratory level (Lab-*Pf*HRP2) (Table 2). This difference was not statistically significant between the 2 groups (p = 0.8848), as well as the number of false negatives was not statistically different between the two groups [1.5% (6/407) versus 1.0% (4/407); p = 0.5209]. However, the number of malaria true negatives was significantly different between HF-*Pf*HRP2 and Lab-*Pf*HRP2 [21.1% (86/407) versus 29.7% (121/407); p = 0.0048], as well as the number of false positives [18.9% (77/407), versus 10.3% (42/407); p = 0.0005] (Table 2).

**Table 2:** Performance of *Pf*HRP2- based rapid diagnostic test performed by study nurses at health facilities (HF-*Pf*HRP2) or *Pf*HRP2-based rapid diagnostic test performed at the central microbiology laboratory (Lab *Pf*HRP2) by trained technicians compared with expert microscopy (gold standard)

Performance characteristic	HF-*Pf*HRP2 n (%)	Lab-*Pf*HRP2 n (%)	p-value
True positive	238 (58.5)	240 (59.0)	0.8848
True negative	86 (21.1)	121 (29.7)	0.0048
False positive	77 (18.9)	42 (10.3)	0.0005
False negative	06 (1.5)	04 (1.0)	0.5209

The sensitivity and specificity of the *Pf*HRP2-RDT supplied by the NMCP and performed by the nurses at health facilities level (HF-*Pf*HRP2) was 97.5% and 52.8%, respectively when using expert microscopy as reference (Table 3). The sensitivity and specificity of the *Pf*HRP2 RDT purchased by CRUN and performed by trained laboratory technicians at laboratory level (Lab- *Pf*HRP2) was 98.4% and 74.2%, respectively (using expert microscopy as gold standard). The positive predictive value of the HF-*Pf*HRP2 was 75.6% and 85.1% for the Lab-*Pf*HRP2 (Table 3). The negative predictive value of the HF-*Pf*HRP2 was 93.5% and that of the Lab-*Pf*HRP2 was 96.8% (Table 3).

**Table 3:** Diagnostic accuracy of *Pf*HRP2 based rapid diagnostic test performed by study nurses at health facilities (HF-*Pf*HRP2) and *Pf*HRP2 based rapid diagnostic test performed at the central microbiology laboratory (Lab *Pf*HRP2) by trained technicians compared using expert microscopy as gold standard

Diagnostic performance characteristic	HF-*Pf*HRP2		Lab-*Pf*HRP2	
	% (n/N)	95% CI	% (n/N)	95% CI
Sensitivity	97.5 (238/244)	94.7–99.1	98.4 (240/244)	95.9–99.6
Specificity	52.8 (86/163)	44.8–60.6	74.2 (121/163)	66.8–80.8
Positive predictive value	75.6 (238/315)	72.4–78.5	85.1 (240/282)	81.5–88.1
Negative predictive value	93.5 (86/92)	86.5–97.0	96.8 (121/125)	91.9–98.8

The agreement between expert microscopy and HF-*Pf*HRP2 was “moderate” (k-value 0.542), that between expert microscopy and Lab-*Pf*HRP2 was “good” (k-value: 0.755) and that between HF-*Pf*HRP2 and Lab-*Pf*HRP2 also “good” (k-value 0.707) (Table 4**)**. Only 49.5% (38/77) of malaria false positives found with HF-*Pf*HRP2 were also tested positive with Lab-*Pf*HRP2 (Tables 2 and 5). However, the remaining 50.5% (39/77) of the false positives found with HF-*Pf*HRP2 were tested negative with Lab-*Pf*HRP2 (Tables 2 and 5). Furthermore, of the 238 malaria true positive cases reported with HF-*Pf*HRP2, 99.6% (237/238) were also tested positive with Lab-*Pf*HRP2 (Tables 2 and 5).

**Table 4:** Agreement between the different diagnostic procedures

	Number of observed agreement n (%)	Number of agreement expected by change n (%)	Kappa (95% CI)	SE of Kappa	Strength of agreement
Field HRP2 and microscopy	324 (79.61)	225.7 (55.45)	0.542 (0.462–0.623)	0.041	Moderate
Lab HRP2 and microscopy	361 (88.70)	219.1 (53.84)	0.755 (0.690–0.820)	0.033	Good
Field HRP2 Lab HRP2	360 (88.45)	246.5 (60.57%)	0.707 (690–0.820)	0.039	Good

**Table 5:** Rapid diagnostic tests results obtained either in the field or in the laboratory compared with expert malaria microscopy findings

	Microscopy + N = 244 n (%)		Microscopy − N = 163 n (%)		Total
	HRP2 + (lab)	HRP2 − (lab)	HRP2 + (lab)	HRP2 − (lab)	
HRP2 + (field)	237 (97.1)	1 (0.4)	38 (23.3)	39 (23.9)	315
HRP2 – (field)	3 (1.2)	3 (1.2)	4 (2.4)	82 (50.3)	92
Total	240	4	42	121	407

## Discussion

Rapid diagnostic tests for malaria are widely implemented by National Malaria Control Programmes in endemic countries, including Burkina Faso, in order to meet the WHO requirement of confirming a malaria infection before starting a treatment [1, 2]. RDTs are hence increasingly replacing (expert) microscopy in many settings, but there is a concern about the diagnostic accuracy of HRP2-based RDTs. Several studies reported a lower sensitivity of RDTs compared to expert microscopy when the parasitaemia is < 200 parasites/µl [24]. This situation is exacerbated by the fact that *Pf*HRP2 polymorphisms are being reported and that certain deletions in this gene may negatively affect RDT performance [25–27]. These *Pf*HRP2 gene deletions have so far not been found in Burkina Faso, but do occur in neighbouring Mali [25]. However, a sensitivity issue was not observed in the present study. There was no significant difference in test sensitivity when the RDT was performed by the two different groups of operators (nurses in the rural health facilities compared to trained laboratory technicians). Importantly, the RDT sensitivity and NPV achieved by both groups almost reached the level of expert microscopy. Only few false negative results were reported with the employed RDTs.

In contrast, the specificity (and subsequently the PPV) of the RDT was worrying low (52.8%) when the test was performed by the nurses at the health facilities level. This is also reflected in the observed agreement between the tests. Overall, the agreement between expert microscopy and Lab-HRP2 was good, but moderate between HF-RDT and expert microscopy. This could be explained by the fact that the HF-RDT was more often false positive. In general, the specificity of the HRP2-based RDTs is being questioned particularly under low transmission conditions [20]. This is supported by one of previous studies conducted in the same study area in which a high prevalence of false positive RDT results was reported during the dry season (April–May; low transmission) [22]. However, the present study was mainly conducted during the rainy season (June–October; high transmission season) but still the number of false positive tests was almost twofold higher at the participating health facilities compared to the laboratory of CRUN. It is more obvious that the number of false positive cases could thus be higher if the study was conducted mainly during the dry season.

HRP2 persistence after a successful treatment is often used as an explanation for the lower specificity of RDTs that are based on the detection of this specific antigen [12–19]. This can however not explain the difference in the test performance observed between the two different groups of operators (i.e. health facility nurses vs laboratory technicians). Several other factors can influence the RDT performance including incorrect test execution and reading of RDT results by health facility nurses whilst performing the test [9, 10]. Also operator errors such as incorrect application of the blood sample or running buffer on the test device, substituting test kit buffer solution with other liquids such as normal saline, diluted water, tap water or buffer from different kits/lots/batches or faulty test devices can affect the test performance by health facility nurses [11]. A very long-reading time could also explain the high positive rate of false positive at health levels. Some non-specific binding or interaction with other immunological or infection factors, such as rheumatoid factors, hepatitis C, schistosomiasis, toxoplasmosis, dengue, leishmaniasis, Chagas’ disease and human African trypanosomiasis can lead to a malaria false positive reaction on a HRP2-based RDT, though considered to be rare [6, 28–36]. It is, therefore, crucial to ensure adequate training of the health workers who perform the RDT and periodically monitor the execution of malaria RDT by the health workers [37].

According to the WHO testing report and the RDT manufacturer information note, the *P. falciparum* specific HRP2-based RDT can stand up to 40 °C for 24 months [6]. However, in Burkina Faso, the mean maximum temperature can reach 45 °C in the dry season [38]. The participating health facilities in this study had no air-conditioning system or temperature and humidity monitoring system in their store room. This can severely affect the test performance as previously reported [8, 39, 40]. Moreover, periodical quality checks of the RDT at the health facilities are not in place. The above-mentioned issues should all be addressed when implementing the malaria *Pf*HRP2 RDT.

Finally, the blood specimen used to perform the malaria RDTs in the field was a capillary sample and the one used to perform malaria RDTs in the central laboratory was from venous blood. It has been reported that the sensitivity of malaria tests (i.e. microscopy) depend on the site of blood collection, in particular in asymptomatic malaria cases. Capillary blood tends to be more sensitive than venous blood [41]. However, the clinical symptoms of malaria infection, including fever, occur in synchrony with the rupture of infected erythrocytes and the release of these erythrocytes and malaria debris in circulating blood [42, 43]. So, it is obvious that symptomatic malaria, which was studied in the present research, will be detectable in capillary blood as well as venous blood.

## Conclusion

Rapid diagnostic tests are a valuable tool for the diagnosis of malaria in settings where expert microscopy is not available. However, some external factors could negatively influence the performance of these RDTs in the field. As long as these factors remain, causes of fever might not be correctly diagnosed and results in inappropriate prescription of anti-malarials and antibiotics in fear of overlooking a treatable infection.

## Abbreviations

CRUN: Clinical Research Unit of Nanoro
EDTA: ethylene diamine tetra acetic acid
FIND: Foundation for Innovative New Diagnostics
HF: health facility
HRP2: histidine-rich protein
Lab: laboratory
LoD: limit of detection
*Pf*: *Plasmodium falciparum*
NMCP: National Malaria Control Programme
NPV: negative predictive value
*p*LDH: *Plasmodium*-specific parasite lactate dehydrogenase
PPV: positive predictive value
RDT: rapid diagnostic test
SD: standard diagnostic
SSA: sub-Saharan Africa
WBC: white blood cells
WHO: World Health Organization
